# Nutritional Stress Leads to Persistence and Persister-like Growth in *Staphylococcus aureus*

**DOI:** 10.3390/pathogens14030251

**Published:** 2025-03-04

**Authors:** Katie R. Risoen, Claire A. Shaw, Bart C. Weimer

**Affiliations:** 100K Pathogen Genome Project, Department of Population Health and Reproduction, School of Veterinary Medicine, University of California, Davis, CA 95616, USA

**Keywords:** persistence, persisters, small colony variants, nutritional stress, bovine mastitis, MRSA, non-culturable

## Abstract

*Staphylococcus aureus* is a versatile zoonotic pathogen capable of causing a wide range of infections. Due to the organism’s ability to persist, recalcitrant and recurring infections are a major concern for public and animal health. This study investigated the establishment of persistence using two *S. aureus* strains—ATCC 29740, a bovine mastitis isolate, and USA300, a human clinical isolate—under substrate depletion. This nutritional stress established a persistence phenotype where the strains remained persistent for >120 days at notable concentrations [>2 log10 CFU/mL] and developed persister-like growth, including small colony variant formations. With RT-qPCR, we found the cell density was higher than represented by the plate count while the intracellular ATP remained constant during the persistence phase. These findings indicate that *S. aureus* has complex survival strategies to support its persistent state, providing a host-specific perspective when addressing recurrent infections in human and animal infectious diseases.

## 1. Introduction

*Staphylococcus aureus,* a ubiquitous gram-positive bacterium, is a highly adaptable, versatile pathogen [1]. While *S. aureus* is a common commensal of the human and animal microbiome, it can cause a wide spectrum of infections in a diverse range of hosts, including livestock, wildlife, and humans [2,3]. In humans, *S. aureus* can range from mild skin and soft tissue infections to severe, life-threatening diseases such as pneumonia, endocarditis, sepsis, and toxic shock [4]. It is one of the leading causes of nosocomial infections and a major contributor to mortality in hospitals [4], fueled by the rise of methicillin-resistant *S. aureus* (MRSA) [5,6] and its capacity to form biofilms [7]. In veterinary medicine, *S. aureus* causes great economic losses in livestock production, most prominently in dairy production, being the leading cause of bovine mastitis worldwide [2,8]. Infections are also widespread in other animals, including swine, poultry, equine, small ruminants, and companion animals [2,9]. Due to the plethora of animal hosts this zoonotic pathogen infects, host-switching events are a common and significant issue at the human/animal interface [9]. For example, more recent zoonotic concerns are being raised as cases of MRSA strains, such as the MRSA ST398 type, which can be transmitted between humans and animals [2,10,11,12]. This opportunistic pathogen is difficult to prevent and treat due to its arsenal of virulence factors that may allow it to survive for prolonged periods in stressful conditions, such as nutritional stress. *Staphylococcus* commonly experiences nutrient-deficient conditions in terrestrial, clinical, and host environments [13]. In addition to the immune system and other harsh host environments, infecting and persisting in a host specifically requires overcoming challenges like nutritional deprivation, such as nutritional immunity and microbial competition [14,15]. Frequent exposure to stresses like this can motivate the development of persistence factors that address nutrient availability or the lack thereof. These traits highlight the ability of *S. aureus* to be a highly adaptive pathogen that quickly acquires antibiotic resistance mechanisms through mobile genetic elements or chromosomal mutations. Such developments were witnessed with the spread of MRSA strains into communities worldwide and now leading to the serious threat of vancomycin-resistant *S. aureus* (VRSA) [16,17,18]. Even if treatment is successful against this adaptive pathogen, *S. aureus* produces heat- and proteolytic-resistant exotoxins that enable further disease to spread after cell death; a noteworthy example is the pyrogenic toxin superantigens that cause toxic shock syndrome and *Staphylococcus*-associated food-borne illness [19].

While virulence factors like the ones detailed above are familiar in the field, recently recognized and understudied persistence mechanisms are found in *S. aureus*, including the phenotypic subpopulations of persisters and small colony variants (SCVs). The persister phenotype is comprised of cellular subpopulations that reduce growth and exhibit antibiotic tolerance. Most antibiotics target active growth mechanisms of microbes, so the inhibition of growth in persisters bypasses the functional mechanisms of the antibiotic. Growth resumes once this stress is removed, suggesting that alternative approaches are needed to understand antimicrobial resistance in concert with persistence. While it is understood that persisters have an altered metabolism and altered ATP production, the mechanisms to enter this antibiotic-tolerant persister state are not well understood [20,21,22]. Within the broad umbrella of persistence are SCVs, being a specific type of persister with an altered colony morphology. SCV colonies appear 1/10 the size of the parent colony with a rougher texture, where development is associated with an intracellular persistence strategy to reduce the expression of virulence factors and invade host cells. This enables protection from antibiotic therapy and cover from the host’s innate defense system [23]. SCVs of *S. aureus*, considered part of the normal bacterial life cycle, can persist within host cells for years and act as reservoirs for chronic infections [23,24,25,26,27]. Mounting clinical evidence is increasingly correlating chronic infections to persisters and SCVs subpopulations, which has led to a surge of research focused on understanding the underlying mechanisms behind their induction and survival strategies and kickstarting the development of anti-persister drugs [20,21,24,28]. For example, SCVs and persisters have been associated with chronic bovine mastitis [29,30], providing an explanation for symptoms such as biofilm development and antibiotic tolerance [8], directing avenues for future medical perspectives and research.

As *S. aureus* continues to be a significant concern to public and animal health, understanding the growth patterns and persistence of *S. aureus* strains from different hosts under stress conditions is important for the development of targeted strategies to prevent, manage, and treat infections in a zoonotic pathogen. The aim of this work was to examine the growth patterns and persistence of two strains under nutritional duress, one isolated from bovine mastitis and another from a human clinical infection. We hypothesized that the two isolates would have different characteristics leading to and during persistence.

## 2. Materials and Methods

### 2.1. Strains and Culture Conditions

*Staphylococcus aureus* ATCC 29740 and USA300 are a bovine mastitis and human clinical isolate, respectively. Both strains were cultured from stock onto Brain Heart Infusion (BHI) plates and incubated at 37 °C for 24 h. One colony of the overnight plate was inoculated into 10 mL of BHI broth (BD Difco, Franklin Lakes, NJ, USA) and incubated overnight at 37 °C, after which the overnight BHI cultures in the exponential phase were all normalized to OD_595_ = 0.1–0.3 using sterile phosphate buffered saline (PBS) to resuspend before inoculation.

### 2.2. Growth Curves in Different Media

Overnight cultures were inoculated at 1% *v*/*v* into BHI broth, Chemically Defined Media (CDM) [31] with 0.5% lactose (Fisher Scientific, Waltham, MA, USA), CDM with 0.5% glucose (Fisher Scientific, Waltham, MA, USA), or Dulbecco’s Modified Eagle Medium (DMEM) (Cytiva, Logan, UT, USA). BHI was used as it is a standard media for culturing *S. aureus*. CDM was selected due to its well-defined composition and ability to control carbohydrate sources, such as glucose and lactose. Glucose is a simple sugar and primary energy source, while lactose is a prominent milk-related sugar relevant to the mastitis isolate.

To generate a growth curve, a 200 µL sample of each inoculated media (BHI, CDM with 0.5% glucose, CDM with 0.5% lactose, and DMEM) was added to a 96-well plate in three technical replicates. The 96-well plate with the inoculated media and corresponding blanks was placed in a Molecular Devices DTX 880 Multimode Detector (Beckman Coulter, Brea, CA, USA) at 37 °C with readings taken every 30 min for 75 h. The plate was grown statically except for 10 s of lateral shaking immediately before each reading.

### 2.3. Nutritional Deprivation and Cell Enumeration

Overnight inoculums of *S. aureus* (10 mL) were incubated for 14 h in BHI and then centrifuged (5000× *g* 5 min). The supernatant was discarded and replaced with an equal volume of PBS to remove BHI nutrients from the initial inoculum. Then, 2 mL of the PBS-resuspended culture was added into three separate bottles of 200 mL CDM, each with 0.5% lactose, and then incubated at 37 °C without shaking. At each time point, for sample collection, 100 μL was taken from each of the three bottles for analysis. Collections occurred at the same time each day (on a 24 h cycle), and serial dilutions for plate counting were done in sterile PBS. BHI plates were divided in half, plating 10 μL of a diluted sample per side of the plate. Growth plating assays were incubated at 37 °C, and colonies were counted at 24 and 48 h of incubation on the plate. This procedure was done at (T) 0, 0.5 (12 h), 1, 2, 3, 4, 5, 6, 7, 8, 9, 10, 14, 16, 21, 23, 50, 70, 87, and 120 days.

### 2.4. Quantitative PCR and ATP Assays

Cell pellets at T_87_ and T_120_ were collected (1 mL) and centrifuged (5000× *g* 5 min) for RT-qPCR and ATP assays. The cell pellet was resuspended in an equal volume of PBS for ATP measurement or 750 μL of TRIzol LS (Invitrogen, Carlsbad, CA, USA) for RT-qPCR analysis. ATP was measured using a BacTiter-Glo Microbial Cell Viability Assay kit (Promega, Madison, WI, USA) according to manufacturer’s instructions, using a DTX 880 Multimode Detector (Beckman Coulter, Brea, CA, USA). 

RNA was extracted as described previously using TRIzol LS [32] and checked for quality and concentration using a NanoDrop (Thermo Scientific, Waltham, MA, USA). From the RNA sample, cDNA was produced using the First Strand cDNA Synthesis (Quick Protocol) (New England Biolabs, Ipswich, MA, USA).

The cDNA was used for RT-qPCR quantification of viable cells using a CFX 96 Real Time System (BioRad, Hercules, CA, USA), according to previously established methods [33]. Briefly, 2x iQ Sybr Green Mastermix (Biorad, Hercules, CA, USA) was used along with 100 nM of forward (F) and reverse (R) PCR primers for the EF-TU gene (F: 5′-ACG CGG 313 TAT CAT CAA AGT GG-3′; R: 5′-ATC GGG TGG ATC AGG GTA AC-3′) to quantify the CFU/mL of cells at T_87_ and T_120_. The parameters for both primers was done using a denaturation step at 95 °C for 5 min, followed by 40 cycles of denaturation, annealing, and extension at 95 °C for 15 s, 56 °C for 30 s, 72 °C for 30 s, respectively, and a final extension at 72 °C for 1 min. The amplified product was verified using melt curve analysis from 50˚C to 95 °C with a transition rate of 0.2 °C/s. To convert cycle threshold (ct) values to CFU/mL, a standard curve was generated from both *S. aureus* USA 300 and *S. aureus* ATCC 29740 using 10-fold dilutions of cDNA, starting with associated plate counts from the extraction material. The standard curves and associated equations of each isolate were then generated using the built-in capabilities of the BioRad CFX Manager qPCR software (version 3.1, BioRad, Hercules, CA, USA). The ct values for technical replicates were then averaged and plugged into the standard curve of the matching isolate to generate an estimated CFU/mL for each biological replicate.

### 2.5. Statistical Analysis

Plate count data are represented as the mean ± SEM of three biological replicates. A Mann–Whitney U Test was used to identify statistically significant differences in plate counts between strains at each time point using R (version 4.0.5). Two-tailed *t*-tests with unequal variances were done using GraphPad Prism 10 (Graphpad, Menlo Park, CA, USA). Growth rate determination in the exponential phase was calculated by finding the best fit third-order polynomial in GraphPad Prism 10. The second-order derivative of each best-fit equation was then used to find the inflection point, with four points around the inflection used to calculate slope and, thus, growth rate. Lag phase calculations were made with the Microbial Lag Phase Duration Calculator with default settings [34]. All figures were visualized with the programs GraphPad Prism 10 and bioRender (bioRender, Toronto, ON, Canada).

## 3. Results

### 3.1. Persistence Establishes Within 72 h in Nutrient Deficient Conditions

**Figure 1:**
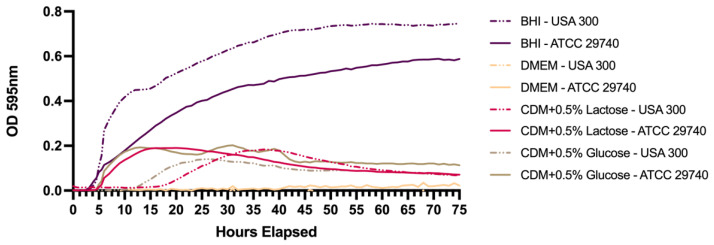
*Staphylococcus aureus* growth curves in different media. Similar cell densities were reached for both strains when grown in media other than BHI. Growth curves in DMEM maintained an OD_595_ of about 0.

To determine the growth patterns, including the length of lag phase and time to stationary phase in different media, ATCC 29740 and USA300 initial growth curves were generated for different media with varying nutrient availability (Figure 1). BHI was used as the positive control for growth analysis, noting that each strain had significantly different growth characteristics that were used to determine individual growth differences within each strain. Within CDM, the maximum density was similar across strain and sugar addition. Furthermore, the rapid decline in optical density reflects the death of susceptible cells and the following selection for persistent subpopulations. Grouping differences were seen between strains rather than between sugar composition. *Staphylococcus aureus* ATCC 29740 had shorter lag phases compared to *S. aureus* USA300, initiating exponential growth a few hours before USA300. While growth rates were similar (Table A1 and Table A2) across strains and media, the lag phase was significant between strains in the same media with lactose sugar (*p* = 0.0033), while glucose sugar was bordering significance (*p* = 0.0871). Insignificant growth was seen when cultured in DMEM.

### 3.2. Persistence Continues for over 120 Days When Challenged with Nutritional Stress and Induces Persister-like Growth Patterns

**Figure 2:**
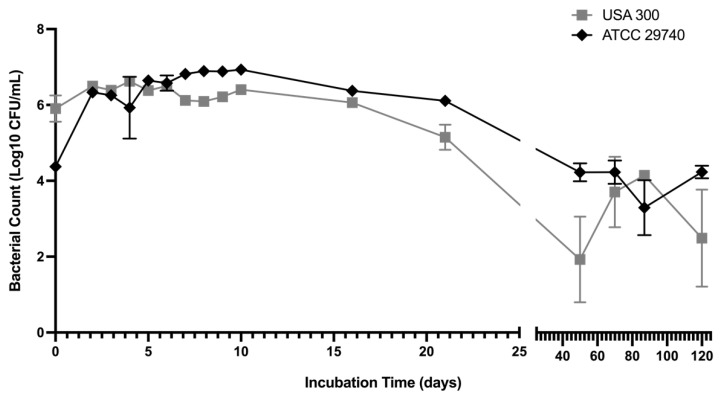
Plate count enumeration during extended incubation in CDM containing 0.5% lactose. Plate counts were averaged over three biological replicates and three technical replicates. Error bars represent ± SEM.

Active replication was determined using plate counts on BHI agar, where persistence was observed over a 120-day incubation (Figure 2). The growth rate of the two strains was not significantly different (*p* > 0.05) within the initial 5 h. Both strains maintained a similar population by CFU/mL plate counts to ~T_7_. Within each strain, the CFU/mL counts remained relatively steady; however, a significant difference (*p* < 0.05) in population between strains was observed during T_7_ to T_10_. With continued incubation, a differential decline in the replicating population was observed.

**Figure 3:**
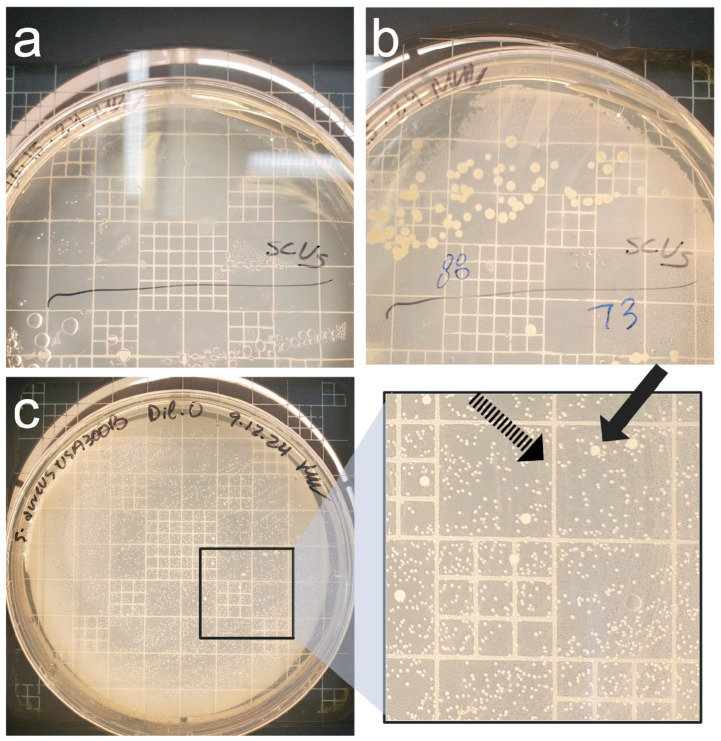
Colony morphology within the culturable population during growth on plating; (**a**) *S. aureus* ATCC 297490 A at T_120_ after 24 h incubation on the plate. There are minuscule colonies appearing, but they are too small to retrieve an accurate count. This characterizes the entire plated population; (**b**) the plate from (**a**) after 48 h of incubation on the plate, showing colonies now have reverted and display wild-type features. (**c**) *Staphylococcus aureus* USA300 B at T_87_ after 24 h of incubation on the plate that contained two phenotypic colonies, wild-type and small morphology, visualized on the zoomed in panel. The dashed arrow indicates SCV-like colonies, while the solid arrow indicates wild-type colonies.

The replicating population continued to differentiate after T_15,_ which included the initiation of three growth characteristics and multiple colony types. The first and most common colony type was ‘wild-type’ growth, with colonies appearing within 24 h with the wild-type colony morphology of *S. aureus*. The second colony type had a smaller colony morphology and appeared within 24 h, exhibiting SCV characteristics. The third observed growth variation was marked by delayed visibility, requiring 48 h of incubation to produce visible colonies. These slow-growing colonies would appear dispersed among wild-type colonies with continued incubation on the plates. Despite the initial delay in appearance on the plates, these colonies would continue growing and become indistinguishable from the wild-type morphology after 48 h (Figure 3). Although there were multiple biological replicates per strain, there were divergences within replicates regarding the occurrence of these three growth patterns, indicating changing subpopulations within each bottle. The appearance of all colony types after long-term incubation in each bottle suggests that each morphology is a persister irrespective of appearance on plates, and perhaps further suggesting different adaptations to the nutritional stress per cell.

### 3.3. Culture-Based Methods of Detection Do Not Accurately Reflect Viability

**Figure 4:**
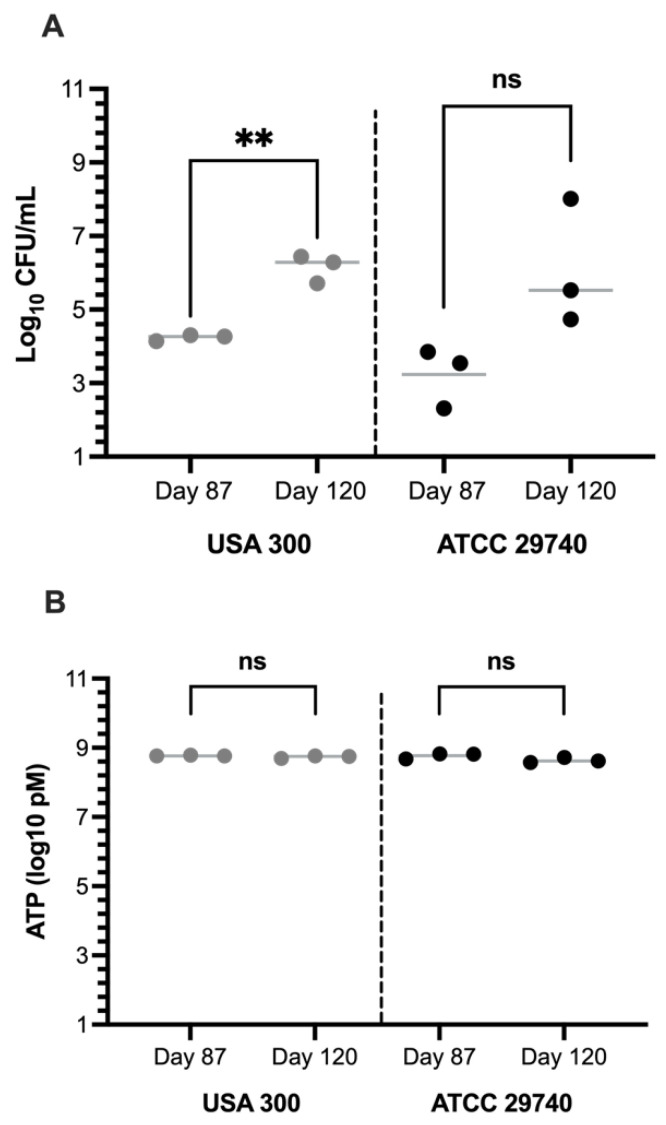
Cell density and intracellular energy during the persistence phase. (**A**) RT-qPCR determining cell viability through EF-TU quantification (refer to Section 2) at T_87_ and T_120_, illustrating an increase in population over these two days. A significant difference was seen between USA300 between T_87_ and T_120_ at *p* = 0.0011. (**B**) ATP assay on T_87_ and T_120_, indicating a maintained composition level of intracellular ATP. ns represents nonsignificant, ** represents *p*-value < 0.005.

To verify the cell viability of the population, RT-qPCR and ATP assays were done at various incubation times during persistence (Figure 4). Results from the qPCR revealed an increase in cell density (Log_10_ CFU/mL) between the two time points with both strains. This result is divergent from the estimate determined using plate counts, suggesting there are two populations in the broth, (1) one that is persistent and (2) one that is non-culturable on plates but intact and metabolically active. Previous work on *S. aureus* and other pathogens supports the existence of mixed populations within persister-inducing conditions, with one subset of cells maintaining the hallmarks of active replication and another existing as non-replicating by viable cells [35,36]. The non-replicating cells, termed viable but non-culturable (VBNC), do not appear on plate counts but continue producing enough ATP for active transport and maintaining intact membranes and metabolic activity [35]. There was a statistical difference seen within USA300 between days T_87_ and T_120,_ but no difference was seen within ATCC 29740 or between strains. To determine if the amount of energy is different between the populations, we used intracellular ATP assays, which remained consistent for both time points, with an average of 0.94 (±0.004) pM ATP or 0.001 (±3.91) pM ATP/CFU. For reference, *S. aureus* grown in nutrient-rich conditions has approximately 0.04 pM ATP/CFU [37]. There were no statistical differences between strains or days.

## 4. Discussion

In this study, we aimed to characterize the persistence and adaptability of *Staphylococcus aureus* strains under conditions of nutritional stress, with significant implications for understanding the mechanisms behind chronic and recalcitrant infections. Furthermore, adopting a multi-host perspective captures the diversity of a pathogen that displays host specificity and specialization, an important factor when developing targeted strategies to prevent and manage persistent infections in both human and veterinary contexts.

When first determining initial growth patterns and the divergence toward persistence, persistence induction remained universal in both strains, becoming steady in the stationary phase within 72 h. This highlights the swift response and adaption to environmental stresses this pathogen is capable of. Considering the short period between inoculation and the induction of persistence, persistence is not an acquired genetic resistance but an innate phenotypic change conserved across *S. aureus* strains in response to stressors. Additionally, persistence within 72 h implies a limited window for effective antimicrobial intervention. Treatments may need to be aggressive and rapid to prevent the formation of persistence phenotypes; however, further studies involving host models will be necessary to see if such rapid treatment would prevent chronic infection altogether.

The difference in the lag phase between the strains could allude to accustomed metabolism plasticity and switching based on the host environment. *S. aureus* derived from bovine mastitis has undergone gene acquisition, loss, and diversification in order to distinctively thrive in the mammary gland [8], expanding its nutrient utilization to incorporate lactose uptake, a common sugar found in dairy milk [38]. The human host environment may encourage catabolite repression, where resource optimization would prioritize glucose over lactose [39], accounting for slight differences of growth between the sugars in *S. aureus* USA300. Lack of growth in DMEM implies that this media lacks key nutrients required for both *S. aureus* growth and persistence, highlighting the auxotrophic nature of the bacteria and its need for specific amino acids, vitamins, and other nutrients found abundantly when housed in its host. Overall, despite known host-switching and host specializations, persistence is a maintained virulence factor, indicating its necessity for infection. Based on this information, we decided to use lactose as our sugar for our extended persistence experiment due to it being the largest difference in persistence induction between the two strains.

When investigating the persistence of this pathogen beyond 72 h, *S. aureus* remained actively replicating for over 120 days. While differences were noticed in initial growth patterns, both human clinical and bovine mastitis *S. aureus* isolates shared similar persistence activities when assessed days and weeks after the initial inoculation. Interestingly, on day 87 of incubation, the evaluation of cell density and metabolic potential by qPCR and ATP concentration suggests that the plate counts accurately captured the population density at that time, while the observations on day 120 suggest a divergence. The lack of congruence between the plate counts, qPCR, and ATP measurements at day 120 in both isolates suggests that subpopulations of differing physiological states contribute to the community counts in non-congruent ways. While active and replicating cells, even with markedly slow replication, appear on the plate, non-active but viable cells can contribute to the ATP and qPCR results without adding to the plate count [36,40]. This discrepancy across different methods of measurements suggests that the emergence of subpopulations within one culture bottle, and potentially within a host or environmental reservoir, may lead to inaccurate assessments of *S. aureus* populations when only one screening method is applied. Such findings should be considered when assessing active or recovered infections in multiple host types.

These findings align with previous studies describing the starvation response of *S. aureus.* Other work similarly found persistent bacteria lasting for months and the development of SCVs when grown in glucose- and multi-nutrient-limited media [13,41]. The fluctuating plate counts and variance in colony morphology between biological replicates over time suggest that persistence is influenced by microenvironmental factors, causing stochastic fluctuations in population. These variations may additionally be heightened by the small sample size, and thus, a larger study will be necessary to tease apart molecular mechanisms behind these differences and to apply clinical relevance to them.

One such concern of clinical relevance in *S. aureus* is the ability of persistent populations to withstand antibiotic treatment and then regrow after the antibiotic stress is removed, ultimately leading to relapsing infections [20]. Persister resuscitation is an underdeveloped but advancing issue that is being addressed in the field. The revival of persisters is triggered through chemotaxis systems, sensing nutrient availability within the environment, such as essential amino acids. Secondary messenger proteins like cAMP that are in high concentrations in persisters are reduced, leading to ribosomal resuscitation and following protein synthesis and growth [42]. Furthermore, the slow growth and altered characteristics of SCVs, such as reduced metabolism and increased antibiotic tolerance [43], allow them to evade the immune system and antibiotic treatment and persist longer in nutrient-depleted environments, contributing to chronic and recurrent infections [24]. The growth patterns observed throughout this experiment resemble that of persisters and SCVs. However, further testing would be required to confirm these phenotypes [27]. The simultaneous occurrence of persister-like colonies among wild-type and the resumed growth after 48 h of incubation could indicate a reversion back to the wild-type phenotype when introduced back to a nutrient-rich environment. The appearance of persister-like colonies at different time points across biological replicates and their subsequent reversion is expected, given the unstable and often transient nature of SCVs [25]. Furthermore, SCVs are difficult to culture, contributing to the challenge of researching this persister, but as this study presents and others have suspected, the strategy of continuous culturing is proving to be an effective strategy for the selection of persisters like SCVs [44].

Confirmation of viability using multiple assays revealed inconsistencies within culture-based detection methods. Culture-based methods of detection are considered the ‘gold standard’ when testing for food contamination and food analysis [45]. However, while the results from the plating and qPCR were similar on T_87_, divergences occurred on T_120_, where the qPCR determined a larger viable population than the culture-based method. With the development of persisters, culture-based methods for detection become unreliable due to the slow growth of these persisters. For example, SCVS may take 2–3 days to become visible on a plate [27], not appearing within the incubation period used within the experiment and, therefore, seemingly having a lower viable population. Additionally, the ATP assay revealed a steady level of intracellular ATP between both strains on both days, confirming viability and active metabolism. When the integrity of the membrane is compromised, ATP production is halted, and ATPases will deplete any remaining intracellular ATP [46]. Based on the maintained ATP presence within the cells at both time points, the population may have reached a favored ATP concentration to sustain a persister state, enough to support this chemotaxis sensing necessary for resuscitation. The 48-h incubations in combination with the small sample size and limited sample collections provide a restricted perspective of the true viability of the population, but local conditions are causing stochastic fluctuations in plate counts, persister-like growth, and viability within these assays, further complicating the phenomenon of persistence.

Our objective was to evaluate the persistence mechanisms involved during nutritional stress in a subset of different host-derived isolates, with a specific interest in the induction of persisters. These findings highlight potential broader implications of *S. aureus* persistence mechanisms, especially when considering the known health risks, food safety, and zoonotic potential of *S. aureus*. By revealing how *S. aureus* adapts and survives under prolonged nutritional stress, this research underscores the capacity of this pathogen to persist in livestock and human settings alike, contributing to recalcitrant and recurring infections. Such adaptability not only complicates treatment but also poses a risk for cross-species transmission, which can amplify the spread of resilient strains and potentially contribute to outbreaks. Understanding these persistence strategies is essential for informing effective therapeutic and preventative measures aimed at minimizing the impact of *S. aureus* on both human and veterinary health. Further investigation into the molecular mechanisms driving SCV formation, persistence, and resuscitation when considering environmental stressors and host-specific factors is essential for improving therapeutic approaches to prevent and manage these challenging infections in human and veterinary medicine.

## Data Availability

Data are contained within the article.

## Abbreviation

The following abbreviation is used in this manuscript:

SCV	Small Colony Variant

## Appendix A

**Table A1:** Two-tailed *t*-tests with unequal variances of growth rates in different medias.

Between Strain	Lactose	0.14370278			
	Glucose	0.684679703			
	DMEM	0.902200382			
	BHI	0.396304143			
USA300		Lactose	Glucose	DMEM	BHI
	Lactose	NA			
	Glucose	0.474397606	NA		
	DMEM	0.459986984	0.404311317	NA	
	BHI	0.275856074	0.171969557	0.648222506	NA
ATCC 29740		Lactose	Glucose	DMEM	BHI
	Lactose	NA			
	Glucose	0.686956269	NA		
	DMEM	0.284479808	0.07761123	NA	
	BHI	0.07761123	0.158564251	0.372151583	NA

**Table A2:** Polynomials for growth rate determination in different medias.

	USA300 Lactose	USA300 Lactose	USA300 Lactose	ATCC 29740 Lactose	ATCC 29740 Lactose	ATCC 29740 Lactose	USA300 Glucose	USA300 Glucose	USA300 Glucose	ATCC 29740 Glucose	ATCC 29740 Glucose	ATCC 29740 Glucose	USA300 DMEM	USA300 DMEM	USA300 DMEM	ATCC 29740 DMEM	ATCC 29740 DMEM	ATCC 29740 DMEM	USA300 BHI	USA300 BHI	USA300 BHI	ATCC 29740 BHI	ATCC 29740 BHI	ATCC 29740 BHI
Polynomial: Third Order (Y = A + B × X + C × X^2^ + D × X^3^)																								
Best-fit values																								
A	−0.02955	−0.02211	−0.04404	−0.03076	−0.00408	−0.00111	−0.02581	−0.03726	−0.03781	−0.03469	0.01471	0.005401	−0.003383	−0.01499	−0.00909	0.0002317	−0.01088	−0.01937	−0.008495	−0.06706	0.006125	−0.03296	−0.03929	−0.01938
B	0.01028	0.00604	0.01091	0.01756	0.01414	0.01442	0.0096	0.008371	0.01159	0.01882	0.01215	0.0143	−0.0008398	−0.0001539	0.0008184	−0.0004362	0.0002157	0.002274	0.03717	0.04576	0.03491	0.02802	0.02734	0.01746
C	−0.0001998	−4.69 × 10^−05^	−0.0001984	−0.0004227	−0.0003586	−0.0003699	−0.0001915	−0.000144	−0.0002658	−0.0004149	−0.0003274	−0.000327	1.92 × 10^−05^	9.57 × 10^−06^	−1.52 × 10^−05^	3.05 × 10^−05^	1.19 × 10^−05^	−4.55 × 10^−05^	−0.0005777	−0.0008184	−0.0006166	−0.0003767	−0.0004096	−0.0002194
D	1.03 × 10^−06^	−1.35 × 10^−07^	9.69 × 10^−07^	2.71 × 10^−06^	2.41 × 10^−06^	2.49 × 10^−06^	1.13 × 10^−06^	6.76 × 10^−07^	1.65 × 10^−06^	2.56 × 10^−06^	2.28 × 10^−06^	2.08 × 10^−06^	−1.53 × 10^−07^	−1.01 × 10^−07^	8.03 × 10^−08^	−2.34 × 10^−07^	−1.28 × 10^−07^	2.62 × 10^−07^	3.05 × 10^−06^	4.67 × 10^−06^	3.58 × 10^−06^	1.65 × 10^−06^	2.23 × 10^−06^	8.96 × 10^−07^
95% CI (profile likelihood)																								
A	−0.04944 to −0.009659	−0.04571 to 0.001492	−0.06369 to −0.02439	−0.04600 to −0.01553	−0.02068 to 0.01252	−0.01817 to 0.01595	−0.03908 to −0.01253	−0.05263 to −0.02188	−0.05377 to −0.02186	−0.05573 to −0.01365	−0.006418 to 0.03584	−0.01603 to 0.02684	−0.006616 to −0.0001501	−0.01899 to −0.01099	−0.01359 to −0.004586	−0.004664 to 0.005127	−0.01424 to −0.007522	−0.02343 to −0.01530	−0.03834 to 0.02135	−0.08336 to −0.05076	−0.03117 to 0.04342	−0.06039 to −0.005528	−0.05340 to −0.02518	−0.03421 to −0.004551
B	0.008121 to 0.01244	0.003478 to 0.008601	0.008783 to 0.01305	0.01591 to 0.01921	0.01234 to 0.01594	0.01256 to 0.01627	0.008159 to 0.01104	0.006703 to 0.01004	0.009857 to 0.01332	0.01654 to 0.02110	0.009854 to 0.01444	0.01197 to 0.01663	−0.001191 to −0.0004890	−0.0005882 to 0.0002803	0.0003297 to 0.001307	−0.0009674 to 9.499 × 10^−05^	−0.0001486 to 0.0005799	0.001833 to 0.002716	0.03393 to 0.04040	0.04399 to 0.04753	0.03086 to 0.03896	0.02504 to 0.03100	0.02581 to 0.02887	0.01585 to 0.01907
C	−0.0002590 to −0.0001406	−0.0001171 to 2.342 × 10^−05^	−0.0002569 to −0.0001399	−0.0004681 to −0.0003773	−0.0004081 to −0.0003092	−0.0004207 to −0.0003191	−0.0002311 to −0.0001520	−0.0001897 to −9.819 × 10^−05^	−0.0003133 to −0.0002183	−0.0004776 to −0.0003523	−0.0003903 to −0.0002645	−0.0003908 to −0.0002631	9.538 × 10^−06^ to 2.879 × 10^−05^	−2.350 × 10^−06^ to 2.148 × 10^−05^	−2.860 × 10^−05^ to −1.776 × 10^−06^	1.588 × 10^−05^ to 4.504 × 10^−05^	1.940 × 10^−06^ to 2.193 × 10^−05^	−5.765 × 10^−05^ to −3.343 × 10^−05^	−0.0006666 to −0.0004888	−0.0008669 to −0.0007698	−0.0007277 to −0.0005056	−0.0004584 to −0.0002950	−0.0004516 to −0.0003676	−0.0002636 to −0.0001753
D	5.783 × 10^−07^ to 1.473 × 10^−06^	−6.660 × 10^−07^ to 3.955 × 10^−07^	5.275 × 10^−07^ to 1.411 × 10^−06^	2.364 × 10^−06^ to 3.049 × 10^−06^	2.032 × 10^−06^ to 2.778 × 10^−06^	2.108 × 10^−06^ to 2.876 × 10^−06^	8.323 × 10^−07^ to 1.429 × 10^−06^	3.305 × 10^−07^ to 1.022 × 10^−06^	1.292 × 10^−06^ to 2.010 × 10^−06^	2.090 × 10^−06^ to 3.036 × 10^−06^	1.802 × 10^−06^ to 2.753 × 10^−06^	1.593 × 10^−06^ to 2.557 × 10^−06^	−2.258 × 10^−07^ to −8.040 × 10^−08^	−1.914 × 10^−07^ to −1.143 × 10^−08^	−2.094 × 10^−08^ to 1.816 × 10^−07^	−3.443 × 10^−07^ to −1.241 × 10^−07^	−2.037 × 10^−07^ to −5.273 × 10^−08^	1.709 × 10^−07^ to 3.538 × 10^−07^	2.378 × 10^−06^ to 3.720 × 10^−06^	4.299 × 10^−06^ to 5.032 × 10^−06^	2.740 × 10^−06^ to 4.417 × 10^−06^	1.034 × 10^−06^ to 2.268 × 10^−06^	1.916 × 10^−06^ to 2.550 × 10^−06^	5.627 × 10^−07^ to 1.230 × 10^−06^
Goodness of Fit																								
Degrees of Freedom	92	92	92	92	92	92	92	92	92	92	92	92	92	92	92	92	92	92	92	92	92	92	92	92
R squared	0.6769	0.6625	0.7278	0.8629	0.7557	0.7563	0.7914	0.7442	0.7214	0.8016	0.595	0.6719	0.6505	0.1705	0.2138	0.8226	0.809	0.6781	0.9693	0.9899	0.9265	0.9633	0.9901	0.9758
Sum of Squares	0.0896	0.1262	0.08741	0.05256	0.06238	0.06593	0.03992	0.05353	0.05765	0.1003	0.1011	0.1041	0.002367	0.003627	0.004594	0.005427	0.002552	0.003744	0.2017	0.06016	0.315	0.1704	0.0451	0.04981
Sy.x	0.03121	0.03703	0.03082	0.0239	0.02604	0.02677	0.02083	0.02412	0.02503	0.03302	0.03315	0.03363	0.005072	0.006279	0.007066	0.007681	0.005266	0.006379	0.04683	0.02557	0.05851	0.04304	0.02214	0.02327
